# LOW TRANSLATION DOWNSTREAM OF TOR ACTIVATES MYOGENE EXPRESSION AND ENHANCES HEALTH OF BODY MUSCLE IN C. ELEGANS

**DOI:** 10.1093/geroni/igz038.1353

**Published:** 2019-11-08

**Authors:** Juyoung K Shim, Santina Snow, Amber Howard, Jordan Horrocks, Joshua Kelley, Hussein Sayed, Aric Rogers

**Affiliations:** 1 MDI Biological Laboratory, Bar Harbor, Maine, United States; 2 MDI Biological laboratory, Salisbury Cove, Maine, United States; 3 University of Maine, Orono, Maine, United States

## Abstract

Previous studies reported that DR or low nutrient signaling reduces translation and extends lifespan, while attenuating growth and reproduction. In this study using C. elegans as a model organism, we found that reduced translation in neurons or germ tissues increases survival and primes the HSR against unfolded protein stress in adult animals. Surprisingly, lowering translation in these tissues significantly upregulates transcription of several key muscle maintenance genes, including those encoding the myogenic response factor HLH-1 and heavy myosin chain factor UNC-54 and MYO-3. It also improves muscle maintenance according to motility assays. However, reduced translation in muscle tissue does not activate the same level of myogenic gene expression. Instead, lowering translation in striated body muscle results in increased reproduction and accelerated development. To further investigate, we examined maintenance of muscle health via deconvolution fluorescent microscopy and immunostaining. Using computational analyses involving Fiji ImageJ and MATLAB program, we discovered that lowering translation delays decrepitude in body muscle structure, resulting in improved mitochondrial morphology and increased thermotolerance. Here we report that, combining physiological, molecular, and microscopic technique, muscle is protected during low translation, low TOR signaling, or DR. We hypothesize that results reflect evolutionary responses that preserve function required for foraging during nutrient scarcity and accelerate development during times low energy usage and high nutrient abundance.

